# Supplementary material for: Assessment of utilization of automated systems and laboratory information management systems in clinical microbiology laboratories in Thailand
Source: PLoS One. 2025 Mar 20;20(3):e0320074. doi: 10.1371/journal.pone.0320074 (PMC11925457; doi:10.1371/journal.pone.0320074)
Supplement: S1 Table — Baseline characteristics of 81 public referral hospitals in Thailand, 2022. (DOCX) [file pone.0320074.s001.docx]

**S1 Table. Baseline characteristics of 81 public referral hospitals in Thailand, 2022**

|  | **Total**  **(n=81)** | **Level-A***  **(n=31)** | **Level-S***  **(n=30)** | **Level-M1***  **(n=20)** | **P value** |
| --- | --- | --- | --- | --- | --- |
| **Characteristics of hospitals** |  |  |  |  |  |
| **Bed count, median (IQR, range)** | 450  (290-678,  150-1387) | 743  (641-860,  509-1387) | 416  (346-450,  215-562) | 252  (215-287,  150-340) | <0.001 |
| **Hospital Information System (HIS)** |  |  |  |  |  |
| **Commercial HIS** | **79 (98%)** | **29 (94%)** | **30 (100%)** | **20 (100%)** | **0.34** |
| HOSxP (Bangkok Medical software, Bangkok, TH) | 54 (67%) | 12 (39%) | 23 (77%) | 19 (95%) |  |
| HoMC (Info-d software, Bangkok, TH) | 9 (11%) | 7 (23%) | 2 (7%)0 | 0 (0%)0 |  |
| Others** | 16 (20%) | 10 (32%) | 5 (17%) | 1 (5%)0 |  |
| **Non-commercial HIS** | **2 (2%)** | **2 (6%)** | **0 (0%)** | **0 (0%)** | - |
| In-house database software | 2 (2%)0 | 2 (6%)0 | 0 (0%)0 | 0 (0%)0 |  |
| **Total number of blood culture bottles processed, median (IQR, range)** | 17351  (9531-29404,  (2900-80330) | 32563  (24274-41895,  15129-80330) | 13027  (10444-17700,  6789-29404) | 7332  (5359-9726,  2900-21647) | <0.001 |
| **Blood culture requesting methods** |  |  |  |  |  |
| Electronic plus paper (i.e. double methods) | 62 (77%) | 22 (71%) | 21 (70%) | 19 (95%) | 0.049 |
| Electronic only (i.e. paperless) | 13 (16%) | 5 (16%) | 8 (27%) | 0 (0%) | - |
| Paper-based only | 6 (7%) | 4 (13%) | 1 (3%) | 1 (5%) | - |
| **Labels on blood culture specimens***** |  |  |  |  |  |
| Printed labels with a barcode | 42 (52%) | 18 (58%) | 15 (50%) | 9 (45%) | 0.64 |
| Printed labels without a barcode | 39 (48%) | 13 (42%) | 15 (50%) | 11 (55%) | - |
| **Characteristics of clinical microbiology laboratories** |  |  |  |  |  |
| **Number of laboratory staff during office hours, median (IQR, range)** | 4  (3-7, 1-14) | 8  (6-9, 4-14) | 3  (3-5, 1-6) | 2  (2-3, 1-4) | <0.001 |
| **Software for daily data management** |  |  |  |  |  |
| **Commercial microbiology LIMS** | **71 (88%)** | **28 (90%)** | **26 (87%)** | **17 (85%)** | **0.81** |
| MLAB (Medical and Food Lab, Bangkok, TH) | 61 (75%) | 22 (71%) | 24 (80%) | 15 (75%) |  |
| Others**** | 14 (17%) | 6 (19%) | 4 (13%) | 4 (20%) |  |
| **Other software** | **6 (7%)** | **2 (6%)** | **3 (10%)** | **1 (5%)** | - |
| WHONET | 3 (4%)0 | 1 (3%)0 | 2 (7%)0 | 0 (0%)0 |  |
| In-house database software | 3 (4%)0 | 1 (3%)0 | 1 (3%)0 | 1 (5%)0 |  |
| **None******* | **4 (5%)** | **1 (3%)** | **1 (3%)** | **2 (10%)** | - |
| **Availability of laboratory staff outside office hours** |  |  |  |  |  |
| 24 hours basis, including public holidays | 29 (36%) | 16 (52%) | 5 (17%) | 8 (40%) | <0.001 |
| Available, but not 24-hours basis | 43 (53%) | 15 (48%) | 23 (77%) | 5 (25%) | - |
| None | 9 (11%) | 0 (0%) | 2 (7%) | 7 (35%) | - |
| **Specimen registration at specimen receptions******** |  |  |  |  |  |
| Recording specimen receipt in a LIMS/other software | 49 (60%) | 23 (74%) | 19 (63%) | 7 (35%) | 0.021 |
| Recording specimen receipt in a HIS | 40 (49%) | 18 (58%) | 12 (40%) | 10 (50%) | 0.40 |
| Recording specimen receipt in a laboratory book | 17 (21%) | 4 (13%) | 6 (20%) | 7 (35%) | 0.20 |
| No data recording at this step | 5 (6%) | 0 (0%) | 3 (10%) | 2 (10%) | 0.14 |

LIMS=Laboratory Information Management System. TH=Thailand. Data are presented as median (IQR, range) unless otherwise specified. *Hospital level is defined by the MoPH Thailand. Level-A is Advanced-level referral hospital, Level-S is Standard-level referral hospital, and Level-M1 is Mid-level referral hospital. **Included SSB (n=7; SSB Mobility, Bangkok, TH), PMK (n=3; H LAB, Bangkok, TH), H.I.M PRO (n=2; HIMPRO, Sisaket, TH), Medical 2020 (n=2; Medical Software, Bangkok, TH), e-PHIS (n=1; Abstract Computer, Bangkok, TH) and Panacea Plus (n=1; Top Provider System and Supply, Pathum Thani, TH). ***Label is defined as a label with patient identifiers (e.g. names, surnames and hospital numbers). ****Included LAB PLUS (n=4; LAB PLUS, Nonthaburi, TH), ALLABIS-M (n=3; ALLABIS, Nonthaburi, TH), GEMs (n=3; GEMS Integrate Solutions, Nonthaburi, TH), SmartBact (n=1; SMART INNOVATION, Bangkok, TH), i-LAB (n=1; ISoft Service, Songkla, TH), Lab Care (n=1; Hospital Expert Infotech, Nonthaburi, TH) and Rax Interdiagnostic (n=1; Rax Interdiagnostic, Bangkok, Thailand). Two level-S and two level-M1 hospitals utilized both MLAB and an additional commercial microbiology LIMS for their daily data management. *****Included two hospitals outsourcing both the bacterial identification and AST steps of the blood culture specimens, and two hospitals directly entering blood culture results into the HIS (HOSxP and HoMC) without using any software for data management in their clinical microbiology laboratory. ******Some clinical microbiology laboratories registered specimens at specimen receptions in multiple systems.
