# Supplementary material for: Assessment of utilization of automated systems and laboratory information management systems in clinical microbiology laboratories in Thailand
Source: PLoS One. 2025 Mar 20;20(3):e0320074. doi: 10.1371/journal.pone.0320074 (PMC11925457; doi:10.1371/journal.pone.0320074)
Supplement: S2 Table — (DOCX) [file pone.0320074.s002.docx]

**S2 Table. Utilization of automated systems for blood culture**

|  | **Total**  **(N=81)** | **Level-A**  **(n=31)** | **Level-S**  **(n=30)** | **Level-M1**  **(n=20)** | **P**  **value** |
| --- | --- | --- | --- | --- | --- |
| **Utilization of an automated blood culture incubation system** |  |  |  |  |  |
| **Yes** | **81 (100%)** | **31 (100%)** | **30 (100%)** | **20 (100%)** | **>0.99** |
| Bactec (Becton Dickinson, MD, USA) | 48 (59%) | 20 (65%) | 21 (70%) | 7 (35%) |  |
| BacT/Alert (BioMérieux, Marcy-l'Étoile, France) | 21 (26%) | 10 (32%) | 5 (17%) | 6 (30%) |  |
| RENDER (Render Biotech, Shenzhen, China) | 4 (5%)0 | 0 (0%)0 | 0 (0%)0 | 4 (20%) |  |
| DL (Zhuhai DL Biotech, Zhuhai City, China) | 4 (5%)0 | 1 (3%)0 | 3 (10%) | 0 (0%)0 |  |
| VersaTREK (ThermoFisher Scientific, MA, USA) | 3 (4%)0 | 2 (6%)0 | 0 (0%)0 | 2 (10%) |  |
| Mindray TDR (Mindray Global, Shenzhen, China) | 3 (4%)0 | 0 (0%)0 | 1 (3%)0 | 2 (10%) |  |
| **No** | **0 (0%)** | **0 (0%)** | **0 (0%)** | **0 (0%)** | - |
| Utilizing in-house prepared blood culture bottles | 5 (6%) | 2 (6%) | 2 (7%) | 1 (5%) | >0.99 |
| % of blood culture bottles being incubated using an automated system (median, IQR, range) | 100%  (100-100, 95-100%) | 100%  (100-100,  95-100%) | 100%  (100-100,  96-100%) | 100%  (100-100,  99-100%) | 0.77 |
| Capacity of automated blood culture system, median (IQR, range)* | 400  (240-600,  120-1200) | 700  (600-800, 290-1200) | 360  (240-400, 200-800) | 200  (148-240, 120-480) | <0.001 |
| **Utilization of an automated bacterial identification system** |  |  |  |  |  |
| **Yes** | **61 (75%)** | **30 (97%)** | **21 (70%)** | **10 (50%)** | **<0.001** |
| Automated biochemical identification system | 59 (73%) | 28 (90%) | 21 (70%) | 10 (50%) |  |
| Vitek (BioMérieux, Marcy-l'Étoile, France) | 36 (44%) | 20 (65%) | 10 (33%) | 6 (30%) |  |
| Sensititre (ThermoFisher Scientific, MA, USA) | 16 (20%) | 7 (23%) | 7 (23%) | 2 (10%) |  |
| MicroScan WalkAway (Beckman Coulter, CA, USA) | 9 (11%) | 3 (10%) | 4 (13%) | 2 (10%) |  |
| Phoenix (Becton Dickinson, Sparks, MD, USA) | 2 (2%)0 | 1 (3%)0 | 1 (3%)0 | 0 (0%)0 |  |
| MALDI-TOF MS-based system** | 10 (12%) | 10 (32%) | 0 (0%)0 | 0 (0%)0 |  |
| Bruker Biotyper CA (Becton Dickinson, MD, USA) | 5 (6%)0 | 5 (16%) | 0 (0%)0 | 0 (0%)0 |  |
| Vitek MS (BioMérieux, Marcy-l'Étoile, France) | 3 (4%)0 | 3 (10%) | 0 (0%)0 | 0 (0%)0 |  |
| Autof MS 1000 (Chirus, Hertfordshire, UK) | 2 (2%)0 | 2 (6%)0 | 0 (0%)0 | 0 (0%)0 |  |
| **No***** | **20 (25%)** | **1 (3%)** | **9 (30%)** | **10 (50%)** | - |
| Performing conventional bacterial identification methods | 72 (98%) | 29 (94%) | 28 (93%) | 15 (75%) | 0.095 |
| % of bacterial isolates from blood culture specimens being identified using an automated system (median, IQR, range) | 90%  (5-100,  0-100%) | 99%  (80-100,  0-100%) | 90%  (0-99,  0-100 %) | 3%  (0-95,  0-100%) | <0.001 |
| **Utilization of an automated AST system** |  |  |  |  |  |
| **Yes** | **61 (75%)** | **30 (97%)** | **21 (70%)** | **10 (50%)** | **<0.001** |
| Vitek (BioMérieux, Marcy-l'Étoile, France) | 35 (43%) | 19 (61%) | 10 (33%) | 6 (30%) |  |
| Sensititre (ThermoFisher Scientific, MA, USA) | 21 (26%) | 12 (39%) | 7 (23%) | 2 (10%) |  |
| MicroScan WalkAway **(**Beckman Coulter, CA, USA) | 9 (11%) | 3 (10%) | 4 (13%) | 2 (10%) |  |
| Phoenix (Becton Dickinson, Sparks, MD, USA) | 3 (4%)0 | 2 (6%)0 | 1 (3%)0 | 0 (0%)0 |  |
| **No***** | **20 (25%)** | **1 (3%)** | **9 (30%)** | **10 (50%)** | **-** |
| Performing conventional AST (e.g. disc diffusion method) | 79 (98%) | 31 (100%) | 29 (97%) | 19 (95%) | 0.52 |
| % of bacterial isolates from blood culture specimens being tested for antimicrobial susceptibility using an automated system (median, IQR, range) | 80%  (3-99,  0-100%) | 95%  (80-99,  0-100%) | 50%  (0-99,  0-100%) | 3%  (0-90,  0-100%) | 0.012 |

AST=antimicrobial susceptibility test. Data are presented as median (IQR, range) unless otherwise specified. We categorized blood culture processing into three steps: incubation, bacterial identification, and antimicrobial susceptibility testing (AST). Some hospitals had more than one automated system for each step. *The capacity of automated blood culture incubation system was defined as the total number of blood culture bottles that could be incubated simultaneously within a clinical microbiology laboratory. **Eight hospitals utilized both an automated biochemical identification system and a MALDI-TOF MS-based system, and the other two hospitals utilized only a MALDI-TOF MS-based system. ***Included two hospitals outsourcing both the bacterial identification and AST steps of the blood culture.
