# Supplementary material for: Assessment of utilization of automated systems and laboratory information management systems in clinical microbiology laboratories in Thailand
Source: PLoS One. 2025 Mar 20;20(3):e0320074. doi: 10.1371/journal.pone.0320074 (PMC11925457; doi:10.1371/journal.pone.0320074)
Supplement: S3 Table — (DOCX) [file pone.0320074.s003.docx]

**S3 Table. Data entry methods to and from commercial LIMS**

| **Data entry methods** | **Total**  **(N=81)** | **Level-A**  **(n=31)** | **Level-S**  **(n=30)** | **Level-M1**  **(n=20)** | **P value** |
| --- | --- | --- | --- | --- | --- |
| **Patient data from HIS to a commercial LIMS** |  |  |  |  |  |
| Automatic import | 43 (53%) | 23 (74%) | 17 (57%) | 3 (15%) | <0.001 |
| Manual entry | 28 (35%) | 5 (16%) | 9 (30%) | 14 (70%) | - |
| Not having a commercial microbiology LIMS** | 10 (12%) | 3 (10%) | 4 (13%) | 3 (15%) | - |
| **Blood culture incubation results (i.e. growth and no-growth) from an automated machine** |  |  |  |  |  |
| Automatic import | 11 (14%) | 10 (35%) | 1 (3%) | 0 (0%) | 0.004 |
| Manual entry* | 60 (74%) | 18 (55%) | 25 (83%) | 17 (85%) | - |
| Not having a commercial microbiology LIMS** | 10 (12%) | 3 (10%) | 4 (13%) | 3 (15%) | - |
| **Bacterial identification results (i.e. bacteria species) from an automated machine** |  |  |  |  |  |
| Automatic import | 35 (43%) | 17 (55%) | 15 (50%) | 3 (15%) | 0.003 |
| Manual entry* | 20 (25%) | 10 (32%) | 4 (13%) | 6 (30%) | - |
| Not having a commercial microbiology LIMS or an automated  system for bacterial identification | 26 (32%) | 4 (13%) | 11 (37%) | 11 (55%) | - |
| **AST results from an automated machine**** |  |  |  |  |  |
| Automatic import | 39 (48%) | 21 (68%) | 15 (50%) | 3 (15%) | 0.002 |
| Manual entry* | 16 (20%) | 6 (19%) | 4 (13%) | 6 (30%) | - |
| Not having a commercial microbiology LIMS or an automated  system for AST** | 26 (32%) | 4 (13%) | 11 (37%) | 11 (55%) | - |
| **Final blood culture results from a commercial LIMS to HIS** |  |  |  |  |  |
| **Automatic data import into HIS** | **1 (1%)** | **1 (3%)** | **0 (0%)** | **0 (0%)** | **0.16** |
| **Manual data entry into HIS** | **20 (25%)** | **6 (19%)** | **6 (20%)** | **8 (40%)** | **-** |
| **Generating picture or PDF files and saving files to HIS** | **29 (36%)** | **11 (35%)** | **12 (40%)** | **6 (30%)** | **-** |
| **Web-based applications to display results from LIMS**  **without entering or transferring data into HIS***** | **14 (17%)** | **9 (29%)** | **5 (17%)** | **0 (0%)** | **-** |
| **Paper-based reports without data entry into HIS** | **7 (9%)** | **1 (3%)** | **3 (10%)** | **3 (15%)** | **-** |
| **Not having a commercial microbiology LIMS** | **10 (12%)** | **3 (10%)** | **4 (13%)** | **3 (15%)** | **-** |
| Exporting data from the in-house database software and  importing data into the HIS through an application  programme interface (API) | 1 (1%)0 | 0 (0%)0 | 1 (3%)0 | 0 (0%)0 |  |
| Manual data entry into HIS | 4 (5%)0 | 1 (3%)0 | 2 (7%)0 | 1 (5%)0 |  |
| Generating picture or PDF files and saving files to HIS | 3 (4%)0 | 2 (6%)0 | 1 (3%)0 | 0 (0%)0 |  |
| Web-based applications to display results from LIMS of  outsourced laboratories without entering or transferring data  into HIS** | 2 (2%)0 | 0 (0%)0 | 0 (0%)0 | 2 (10%) |  |

*In case that a clinical microbiology laboratory had more than one automated system for that step and at least one automated system required manual data entry into the commercial LIMS, we classified as ‘Manual entry’ **Included two hospitals outsourcing both the bacterial identification and AST steps of the blood culture. ***Web-based applications included both intranet and internet applications.
