# Supplementary figures and images for: Assessment of utilization of automated systems and laboratory information management systems in clinical microbiology laboratories in Thailand
Source: PLoS One. 2025 Mar 20;20(3):e0320074. doi: 10.1371/journal.pone.0320074 (PMC11925457; doi:10.1371/journal.pone.0320074)

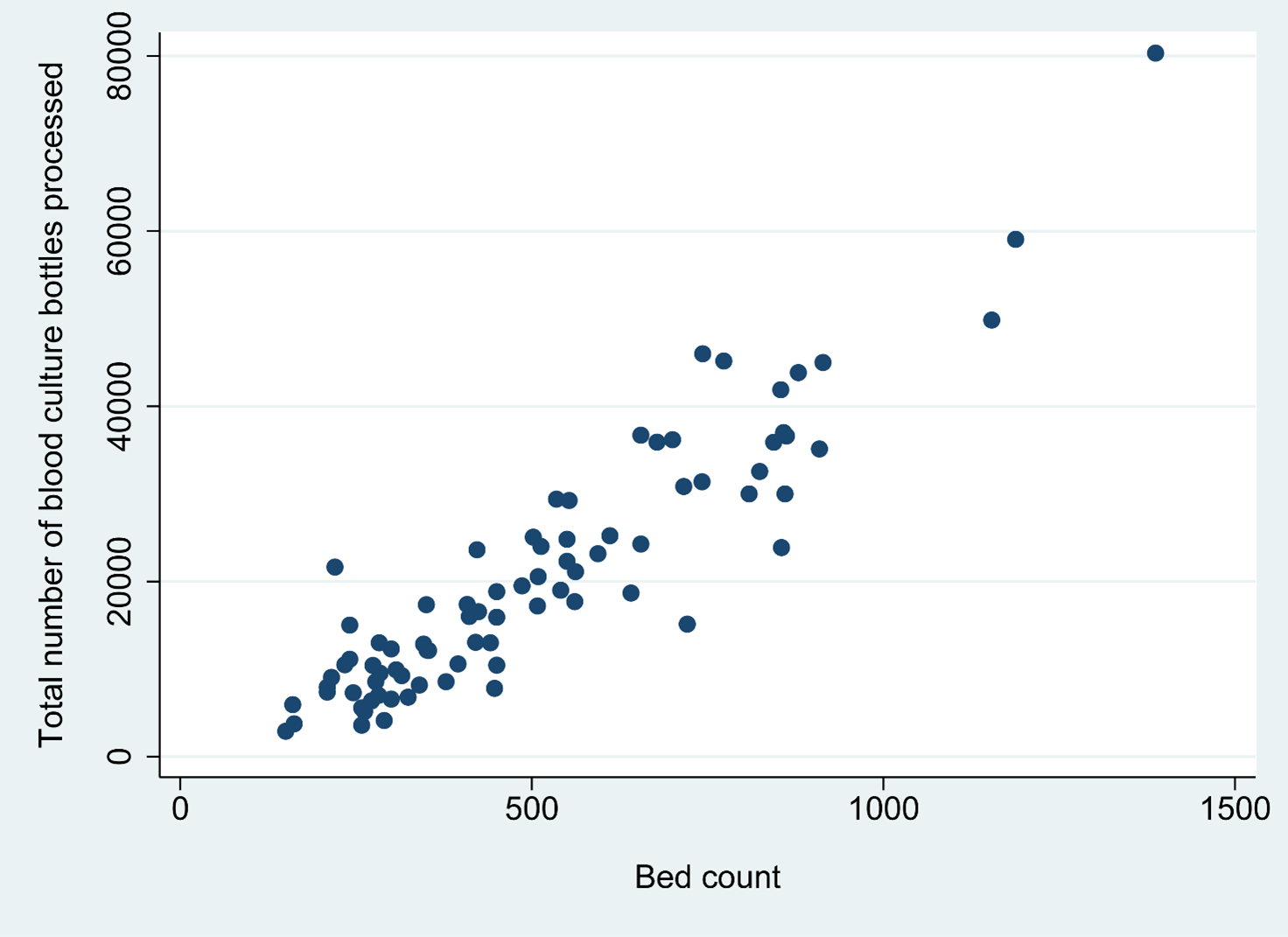

Supplement: S1 Fig — (TIF) [file pone.0320074.s004.tif]
