# Supplementary material for: Assessment of utilization of automated systems and laboratory information management systems in clinical microbiology laboratories in Thailand
Source: PLoS One. 2025 Mar 20;20(3):e0320074. doi: 10.1371/journal.pone.0320074 (PMC11925457; doi:10.1371/journal.pone.0320074)
Supplement: S1 File — (PDF) [file pone.0320074.s005.pdf]

Instructions: Please answer the questions and specify the best answers that match your situations or opinions.

For questions where you can select a single answer, there will be a circular button "O". Please choose the most correct answer. For questions where you can select more than one answer, there will be a rectangular button "☐".

***In this questionnaire, the term "microbiology laboratory" means "microbiology work" and "bacteriology work" for your laboratory, even if your laboratory does not physically or literally separate "microbiology laboratory" as a specialty.**

***In this questionnaire, we generally ask about the capabilities and barriers of blood culture samples collected in your hospital. Blood culture samples sent from other hospitals for bacterial culture in your laboratory will not be included unless specified in the question.**

**1.**	**Part 1 General questions about your hospital**
**1.1**	Which hospital do you work at? (Select one hospital from the 127 hospitals level of A, S and M1 under the Ministry of Public Health [MoPH])
O	(Drop down box - 127 hospital list)<br>***** Please stop answering the questionnaire if you do not work in microbiology or bacteriology at any of the 127 hospitals level of A S M1 under the Ministry of Public Health.**
**1.2**	How many employees work in the microbiology laboratory at your hospital during office hours?<br>|___|___| *** Estimate the number of staff working in the laboratory during the office hours. **<u>Do not include staff who works outside office hours.</u>**<br><br>**<u>If the number of staff is less than one, please answer one and then add details to question 1.12</u>**<br>This could occur when some staff may work on both "microbiology work" and "other laboratory work" (such as biochemistry laboratory work). Please estimate the proportion of laboratory staff actually working on microbiology. For example, 0.5 employee (about 50% of an employee's time during officer hours).
**1.3**	Did a laboratory staff participated in the Antimicrobial Susceptibility testing for AMR training program from the MoPH or other public institutions (You may select more than one answer.)
☐	Yes, Program of Medical Technology Specialty of Clinical Microbiology (Antimicrobial resistant bacteria), organized by Faculty of Medical Technology, Mahidol university and sponsored by Health Administration Divisio
☐	Yes, Program of Medical Technology Specialty of Clinical Microbiology (Antimicrobial resistant bacteria), organized by Faculty of Medical Technology, Mahidol university (MUMT), and sponsored by your hospital or health network
☐	Yes, Program of Medical Technology Specialty of Clinical Microbiology (Antimicrobial resistant bacteria), organized by a public university in Thailand
☐	Yes, the Antimicrobial Susceptibility testing for AMR workshop, organized by NARST (National Antimicrobial Resistant Surveillance Center, Thailand)
☐	Yes, a short course/workshop (3-14 days) for AMR, organized by a public university in Thailand
☐	No personnel trained by public universities
☐	Others, please specify____
**1.4**	Did a laboratory staff repeat the course in 1.3?
☐	The training has been repeated in the past year
☐	Retraining, but not in the past year
☐	Never retrained
**1.5**	In 2024, which course would your laboratory like to attend for diagnosing drug-resistant bacteria?
☐	Yes, Program of Medical Technology Specialty of Clinical Microbiology (Antimicrobial resistant bacteria), organized by Faculty of Medical Technology, Mahidol university
☐	Yes, Program of Medical Technology Specialty of Clinical Microbiology (Antimicrobial resistant bacteria), organized by a public university in Thailand
☐	Yes, the Antimicrobial Susceptibility testing for AMR workshop, organized by NARST (National Antimicrobial

Resistant Surveillance Center, Thailand)
☐ Yes, a short course/workshop (3-14 days) for AMR, organized by a public university in Thailand
☐ Others, please specify______________________________

1.6 In the laboratory, do you want to send personnel to attend a training course on the diagnosis of resistant bacteria? (you can select more than one answer)
☐ Yes, and personnel can be sent to join as soon as 2024.
☐ Yes, but personnel cannot be sent to join in 2024
☐ Not required
☐ Others, please specify______________________________

1.7 What program does your hospital currently use as a Hospital Information System (HIS) to store patient data?
○ HOSxP
○ HoMC
○ SSB
○ Panacia Plus Hospital Solution (PPHS)
○ H.I.M. PRO (Hospital Information Management Professional)
○ Others, please specify ______________________________

1.8 What is the main method that medical staff at your hospital **use when sending a request for blood culture testing** to the microbiology laboratory?
○ Paper-based only
○ Paper plus electronic
○ Electronic only

1.9 What kind of **label is applied to the blood culture sample before** it is sent to the microbiology laboratory?
○ The label is handwritten only.
○ The label is printed but does not have a barcode.
○ The label is printed and has a barcode.
○ There is no label on the blood culture sample to be sent to the microbiology laboratory.

1.10 Does the laboratory staff record any information **when a blood culture sample is received before performing any procedures on the sample?** For example, before placing the blood sample in an incubator for culture. (You may select more than one answer.)
☐ Record the information on the label that is on the container in the **laboratory book** "before" performing any procedures on the sample (e.g., write information by hand or apply a copy of the label in the laboratory book).
☐ Type the information on the label that is on the container into the **Excel program** "before" performing any procedures on the sample.
☐ Type the information on the label that is on the container into **the laboratory software** (such as MLAB, WHONET, ALLABIS-M, and others) "before" performing any procedures on the sample.
☐ Record that the item being tested has been "received" on **the hospital information system** (such as HoMC and HOSxP or others) because medical personnel have ordered it through the hospital information system and the hospital information system already has the initial record of the sample.
☐ Type the information on the label that is on the container into **the hospital information system** (such as HoMC and HOSxP or others) **by starting a new record of the blood culture sample by the laboratory staff.**
☐ **There is no data recording "before" performing any procedures on the sample.** Data recording will begin after some of the procedures have been done (such as recording the bacterial results on paper forms, notebooks or software) only after some or all of the laboratory procedures are completed.
☐ The barcode on the label is scanned (regardless of which data is obtained and used from the barcode).
☐ **Others, please specify**______________________________

1.11 Can your microbiology laboratory **automatically import patient data** (such as name, last name, and HN) **from the hospital information system (such as HoMC and HOSxP)?**
("Automatically" means "There is no need to manually enter patient data (such as name, last name, and HN) into the laboratory software again".)
○ Yes, data from the hospital information system, such as name, last name, and HN, **will automatically appear in the laboratory software** (such as MLAB and ALLABIS-M).
○ **No, it cannot be used.** Laboratory staff must **manually** enter the name, last name, and HN in the laboratory

	software.
○	**Others, please specify** ____________________
**1.12**	Can your microbiology laboratory utilize **the barcode** on the blood culture sample? (You can choose more than one answer.)
☐	**It cannot be used** because there are **no barcodes** on blood culture samples.
☐	**It cannot be used** because the microbiology laboratory cannot utilize **the barcode** on blood culture samples.
☐	**It can be used. By scanning the barcode, the laboratory staff can start recording that the blood culture is received or can start entering culture results into the hospital information system** (such as HoMC and HOSxP) without the need to manually enter patient data (such as name, last name, and HN) in the **hospital information system**.
☐	**It can be used. By scanning the barcode, the laboratory staff can start recording that the blood culture is received or can start entering culture results into the laboratory software (**such as MLAB and ALLABIS) without the need to manually enter patient data (such as name, last name, and HN) in the **the laboratory software.**
☐	**It can be used**. The barcode scanning can be used before placing blood culture samples in automated blood culture incubation systems to connect patient data (such as name, last name and HN)
☐	**It can be used**. The barcode scanning can be used to connect incubation results, bacterial identification results or AST results from automated systems
☐	**Others, please specify** ____________________
**1.13**	Does your microbiology laboratory have staff working on a 24-hour basis?
○	Yes, there are staff working **on a 24-hour basis, including public holidays.**
○	No, there are staff working **only during office hours.**
○	No, there are staff working **on outside of office hours, but not on a 24-hours basis.**
**1.14**	**When there is no one working or on duty** and a blood culture sample arrives at the laboratory, what does your microbiology laboratory do?
○	**Not applicable** since laboratory staff are working on a 24-hour basis, including public holidays.
○	Since the laboratory does not accept blood culture samples outside of office hours, **no blood culture samples arrive outside of office hours.** However, the blood culture sample will be **kept at a room temperature or at a normal temperature in the ward area or examination room.**
○	Since the laboratory does not accept blood culture samples outside of office hours, **no blood culture samples arrived outside of office hours.** However, the blood culture samples will be **kept in an incubator in the ward area or examination room.**
○	**The blood culture samples will be kept at a room temperature or at a normal temperature in an area of the laboratory**
○	**The blood culture samples will be kept in an incubator in an area of the laboratory**
**1.15**	How does your microbiology laboratory do if blood culture samples in an automated blood culture incubation system —such as using BD or BacT/ALERT—show a positive result **when there are no staff working?**
○	**Not applicable** because there are staff working on a 24-hour basis, including on holidays.
○	**Not applicable** since automated blood culture incubation systems are not used.
○	**Waiting until working hours for additional action.**
○	Others, please specify ____________________
**1.16**	**Any remarks on the answers in part 1**

2.	**Part 2 Blood cultures**
**2.1**	**In 2022,** what was the total number of blood culture samples (bottles) sent to your microbiology laboratory? (In this question, the total number includes blood culture samples collected in your hospital and those sent from outside your hospital; such as from other hospitals or other organizations. The total number also includes blood culture samples collected from other activities, such as outbreak surveillance. Please count or estimate the total number of blood culture bottles as precisely as possible.)<br>|___|___|___|,|___|___|___| bottles
**2.2**	**In 2022**, did your microbiology laboratory receive **any blood culture samples from outside your hospital?**
○	Yes
○	No
**2.3**	**In 2022,** what was the total number of blood culture samples (bottles) sent to your microbiology laboratory **from outside your hospital**? **(Please count or estimate the total number of blood culture bottles as precisely as possible.)**<br>|___|___|___|,|___|___|___| bottles<br>*** If there were no blood culture samples sent to your microbiology laboratory **from outside your hospital**, please answer 0 bottles.
**2.4**	What types of culture bottles were used **in your hospital** (excluding blood culture samples from outside your hospitals)? (Responses should range from 1 to 100 percent for each type. If a type of blood culture bottle was not used at all, please answer 0%) **(The total should be 100%.)**<br>|___|___|___| % Manual blood culture bottles.<br>|___|___|___| % BACTEC bottles from BD.<br>|___|___|___| % BacT/ALERT bottles from Biomerieux.<br>|___|___|___| % Others, please specify____
**2.5**	What methods did you use to cultivate blood culture samples in your microbiology laboratory? (Responses should range from 1 to 100 percent of the total number of blood culture samples. If a method was not used at all, please answer 0%) **(The total should be 100%.)**<br>|___|___|___| % Conventional/manual method by tracking the daily growth of bacteria<br>|___|___|___| % Continuously monitored automated system such as BD or BacT/ALERT<br>|___|___|___| % Send the blood culture samples to other laboratories outside your hospital<br>|___|___|___| % Others, please specify____
**2.6**	**How many blood culture bottles can you incubate simultaneously,** if you use an automated system. (For example, if you have two of 400-bottle automated machines, you can cultivate up to 800 bottles simultaneously). (If you did not have an automatic system, please answer 0 bottles)<br>|___|, |___|___|___| bottles
**2.7**	When a blood culture sample is alerted as "**positive" (and bacteria have not yet been identified),** did you record or enter any data? (You may select more than one answer.)
☐	Record "Positive" in the laboratory book immediately and "manually"
☐	Record "Positive" in the Microsoft Excel immediately and "manually"
☐	Record "Positive" int the laboratory software immediately and "manually".
☐	Record "Positive" in the laboratory software "automatically" (without the need to type anything manually)
☐	Record "Positive" in the hospital information system (such as HoMC HOSxP etc.) immediately and "manually".
☐	Record "Positive" in the hospital information system (such as HoMC HOSxP etc.) "automatically" (without the need to type anything manually)
☐	No "Positive" data is entered or recorded at this step because data entry will start only when there is additional information.
☐	Others, please specify ____<br>*** "Automatically" means "There is no need to open the laboratory software (or hospital information system), no need to manually locate records, and no need to manually type or enter result data into the laboratory software (or hospital information system).
**2.8**	When a blood culture sample is finalized as "**negative/no growth",** how did you record or enter the result? (You

	may select more than one answer.)
☐	Record "negative/no growth" in the laboratory book "manually"
☐	Record "negative/no growth" in the Microsoft Excel "manually"
☐	Record "negative/no growth" in the laboratory software "manually".
☐	Record "negative/no growth" in the laboratory software "automatically" (without the need to type anything manually)
☐	Record "negative/no growth" in the hospital information system (such as HoMC HOSxP etc.) "manually".
☐	Record "negative/no growth" in the hospital information system (such as HoMC HOSxP etc.) "automatically" (without the need to type anything manually)
☐	Record "negative/no growth" in the hospital information system (such as HoMC HOSxP etc.) by saving the results from the laboratory software as a picture or PDF, and attaching the picture or PDF to the hospital information system
☐	Others, please specify ______________________________
	*** "Automatically" means "There is no need to open the laboratory software (or hospital information system), no need to manually locate records, and no need to manually type or enter result data into the laboratory software (or hospital information system).
**2.9**	**Any remarks on the answers in part 2**

3. Part 3 Bacterial identification (blood culture samples)

3.1 **<u>In 2022</u>**, how many blood culture samples were processed for 'bacterial identification' at your microbiology laboratory?
(In this question, the total number includes the blood culture sample collected in your hospital and those sent from outside your hospital; such as other hospitals or other organizations**<u>, and processed for 'bacterial identification'.</u>** The total number also includes blood culture samples collected from other activities, such as outbreak surveillance**<u>, and processed for 'bacterial identification'.</u>** Please count or estimate the total number of blood culture bottles as precisely as possible.)
|___|___|___|,|___|___|___| samples
*** This question excludes bacteria identified as "contaminated bacteria" without being processed for 'bacterial identification' (as described in the section 3.2).

3.2 **<u>In 2022</u>**, what methods were used for bacterial identification from blood culture samples? (Response should range from 1 to 100 percent of the total samples tested using each method. If a method was not used at all, please answer 0%) (Please estimate as accurately as possible. **<u>The total may exceed 100% because some samples were tested using multiple methods</u>**.)

|___|___|___| % Gram stain
|___|___|___| % Biochemical test
|___|___|___| % Vitek® 2 (Biomeieux Ltd.)
|___|___|___| % BD Phoenix™ (Becton Dickinson Ltd.)
|___|___|___| % Sensititre™ (Thermo Fisher Scientific Ltd.)
|___|___|___| % MicroScan® ID (OMRON Ltd.)
|___|___|___| % VITEK® MS (MALDI-TOF, Biomeieux Ltd.)
|___|___|___| % BD™ Bruker MALDI (MALDI-TOF, Becton Dickinson Ltd.)
|___|___|___| % Send samples to other laboratories outside the hospital for bacterial identification
|___|___|___| % Others, please specify____

3.3 **<u>In 2022, did you record the results of bacterial identification from blood culture samples in laboratory book</u>** "manually" on a daily basis?

○ Yes
○ No

3.4 **<u>In 2022, on a daily basis, did you use any laboratory software to record blood culture results</u>**? (If the software has changed, please respond using the most recent version.) (This question **<u>does not include</u>** the programmes which you used only for importing data into in order to perform statistical analysis or generate cumulative antibiograms or reports.) (You may select more than one answer.)

☐ MS Excel
☐ MLAB
☐ WHONET
☐ ALLABIS-M
☐ GEMs
☐ SmartBact
☐ Lab-Plus
☐ I-LAB
☐ A local-made program developed based on MS Access
☐ A local-made program that is neither based on MS Excel nor MS Access.
☐ A program was not used because blood culture samples was sent for testing from outside your hospital laboratory.
☐ Others, please specify____

3.5 **In 2022, on a daily basis, which software did you use as your main laboratory software for data storage**? (This question **<u>does not include</u>** the programmes which you used only for importing data into in order to perform statistical analysis or generate cumulative antibiograms or reports.) (You can only choose one answer)

○ MS Excel
○ MLAB
○ WHONET
○ ALLABIS-M

- O GEMs
- O SmartBact
- O Lab-Plus
- O I-LAB
- O A local-made program developed based on MS Access
- O A local-made program that is neither based on MS Excel nor MS Access.
- O A program was not used because blood culture samples was sent for testing from outside your hospital laboratory.
- O Others, please specify____

3.6 **In 2022,** how did you enter "**the results of bacterial identification**" from automated systems into "**your main laboratory software for data storage**" ?

Vitek® 2	O "Manually"	O "Automatically"	O This machine doesn't exist.
BD Phoenix™	O "Manually"	O "Automatically"	O This machine doesn't exist.
Sensititre™	O "Manually"	O "Automatically"	O This machine doesn't exist.
MicroScan® ID	O "Manually"	O "Automatically"	O This machine doesn't exist.
VITEK® MS	O "Manually"	O "Automatically"	O This machine doesn't exist.
BD™ Bruker MALDI	O "Manually"	O "Automatically"	O This machine doesn't exist.

*** "Automatically" means "There is no need to open the laboratory software, no need to manually locate records, and no need to manually type or enter result data into the laboratory software.

3.7 **Any remarks on the answers in part 3**

4.	**Part 4 Antimicrobial susceptible testing (blood culture samples)**
4.1	**<u>In 2022, how many</u>** blood culture samples were tested for AST **in your microbiology laboratory?**<br>(In this question, the total number includes blood culture samples collected in your hospital as well as those sent from outside sources, such as other hospitals or organizations, and tested for AST. It also includes blood culture samples collected from other activities, such as outbreak surveillance, and tested for AST. Please count or estimate the total number of blood culture bottles as precisely as possible.)<br>|___|___|___|, |___|___|___| samples
4.2	**In 2022,** what methods did your microbiology laboratory use for AST testing of blood culture samples? (Response should range from 1 to 100 percent. If a method was not used at all, please answer 0%. Please estimate as accurately as possible. **<u>The total may exceed 100% because some samples were tested using multiple methods</u>**.)<br>|___|___|___| % Disk diffusion test or Kirby Bauer's method<br>|___|___|___| % MIC determination by broth dilution<br>|___|___|___| % MIC determination by E-test<br>|___|___|___| % Broth microdilution (96-well tray)<br>|___|___|___| % Broth microdilution (tube method)<br>|___|___|___| % Agar dilution<br>|___|___|___| % Vitek® 2 AST (Biomeieux Ltd.)<br>|___|___|___| % BD Phoenix™ AST (Becton Dickinson Ltd.)<br>|___|___|___| % Sensititre™ AST (Thermo Fisher Scientific Ltd.)<br>|___|___|___| % MicroScan® AST (Beckman Coulter Ltd.)<br>|___|___|___| % SIRscan ORION (I2a Ltd.)<br>|___|___|___| % Sent samples to other laboratories outside the hospital for AST testing<br>|___|___|___| % Others, please specify____
4.3	**<u>In 2022, did you record the AST results from blood culture samples in the laboratory book</u>** "manually" on a daily basis?
○	Yes
○	No
4.4	**<u>In 2022,</u>** how did you enter "**the AST results"** from automated machines into "**your main laboratory software for data storage"** ?<br>Vitek® 2 AST ○ "Manually" ○ "Automatically" ○ This machine doesn't exist.<br>BD Phoenix™ AST ○ "Manually" ○ "Automatically" ○ This machine doesn't exist.<br>Sensititre™ AST ○ "Manually" ○ "Automatically" ○ This machine doesn't exist.<br>MicroScan® AST ○ "Manually" ○ "Automatically" ○ This machine doesn't exist.<br>SIRscan ORION ○ "Manually" ○ "Automatically" ○ This machine doesn't exist.<br>*** <u>"Automatically"</u> means "There is no need to open the laboratory software, no need to manually locate records, and no need to manually type or enter result data into the laboratory software.
4.5	**<u>In 2022,</u>** after completing bacterial identification and AST results, did you record or enter any data into the **hospital information system** (such as HoMC and HOSxP **)**?
○	No, because the hospital information system cannot display the blood culture results.
○	No, because the hospital information system can "automatically" retrieve blood culture results from the laboratory software (such as MLAB and ALLABIS-M).
○	**Yes, laboratory staff have to manually record or enter <u>some</u> data** into the hospital information system while some data, such as the name of the bacteria species, were not need to be entered.
○	**Yes, laboratory staff have to manually record or enter <u>all</u> data** into the hospital information system
○	**Yes, by saving results as a pictures or PDF, and attached a pictures or PDF** into the hospital information system
○	Others, please specify ____<br>*** <u>"Automatically"</u> means "There is no need to open the hospital information system, no need to manually locate records, and no need to manually type or enter result data into the hospital information system.
4.6	**<u>In 2022</u>**, when an AMR bacteria under the surveillance was observed, did your laboratory have a Lab Alert system to relevant parties (e.g. AMS, IPC team)? (You can select more than one answer)

- ☐ **No Lab Alert**
- ☐ Notify relevant parties via telephone immediately.
- ☐ Notify relevant parties by sending a picture of the culture result report via the LINE application.
- ☐ Notify relevant parties by sending a file of culture result report via LINE application.
- ☐ Notify relevant parties and have a system to link the data of culture results with a drug prescription/administration system so that it can adjust antibiotic based on AST results within 24 hours
- ☐ Notify via the laboratory software (e.g. ALLABIS, MLAB or others), because there is a notification system or notification message when AMR bacteria under the surveillance was observed detected. Stakeholders can access the results through the hospital information system (e.g. HoMC HOSxP or others).
- ☐ Notify by entering data into a programme for IC nurses/teams of the hospital
- ☐ Notify via paper-based only
- ☐ Notify via the hospital information system (such as HoMC and HOSxP) only
- ☐ Notify via both paper-based and the hospital information system (such as HoMC and HOSxP)
- ☐ Others, please specify ____

4.7	**Any remarks on the answers in part 4**

5.	**Part 5 Data analysis (blood culture samples)**
**5.1**	Did your microbiology laboratory generate **a cumulative antibiogram for the year 2022**? (You may select more than one answer.)
☐	No
☐	Yes, a cumulative antibiogram report for the year 2022 was created using **the software for laboratory data storage.**
☐	Yes, a cumulative antibiogram report for the year 2022 was created using the **WHONET**
☐	Yes, a cumulative antibiogram report for the year 2022 was created using the **AMASS** via the workshop organized by the Health Administration Division, MoPH, Thailand
☐	Others, please specify ____
**5.2**	Did your microbiology laboratory **import data of the year 2022 into WHONET for data analysis in order to send the WHONET data to the Department of Medical Science, MoPH.** (You may select more than one answer.)
☐	No, our microbiological laboratory did not use the **WHONET**.
☐	No, our microbiological laboratory recorded data into the **WHONET on a daily basis**. Therefore, we did not have to import data into WHONET.
☐	I attempted but **failed** to import data into the WHONET.
☐	**Yes. However, our microbiology laboratory has not used the data in the WHONET yet**
☐	**Yes. Our microbiology laboratory used the data in the WHONET to generate a cumulative antibiogram**
☐	**Yes. Our microbiology laboratory used the data in the WHONET for data analysis (such as quality control and outbreak analysis)**
☐	**Yes. Our microbiology laboratory sent the data to the Department of Science in the WHONET format**
☐	I exported data from the **major data storage software** and **emailed** the file to the Department of Science.
☐	I exported data from the **major data storage software** and sent the file to the Department of Science through the **ALISS system.**
☐	Others, please specify ____
**5.3**	Do you think whether the data of blood culture samples of the year 2022 stored **in the main laboratory software was complete or not**?
☐	Incomplete. Only positive bacterial culture results were available. (Negative or no growth results were not stored in **the main laboratory software**.)
☐	Incomplete. Some **bacterial identification data** was only stored in some automated machine and has not yet been transferred, stored or entered into **the main laboratory software**.
☐	Incomplete. Some **AST data** was only stored in some automated machine and has not yet been transferred, stored or entered into **the main laboratory software**.
☐	Incomplete. Some data, such as HN (hospital number), in **the main laboratory software** does not match with the data in the hospital information system. The total amount of errors may cause misinterpretation of data analysis (such as a cumulative antibiogram).
☐	Incomplete. Some data, such as HN (hospital number), in **the main laboratory software** does not match with the data in the hospital information system. The total amount of errors is negligible and will not cause misinterpretation of data analysis (such as a cumulative antibiogram).
☐	100% complete.
☐	Others, please specify ____
**5.4**	For the data of year 2022, did you have any problems when **exporting** blood culture sample data from the main laboratory software? (You may select more than one answer.)
☐	Yes. The data exported from the **main laboratory software** was incomplete.
☐	Yes. Data were stored in more than one laboratory software, making it difficult or impossible to merge or export the data.
☐	Yes. Laboratory software was changed during the year, making it difficult or impossible to merge or export the data.
☐	There were no problems.
☐	Others, please specify ____
	*** The problem of data analysis and generating of a cumulative antibiogram by microbiology laboratories is

common in Thailand and other low and middle-income countries. Having problems does not imply that the work of microbiology laboratories is inadequate. **Your answers would help MoPH understand real-world problems, enabling them to support and provide solutions in the future.**

5.5 **Which problems did you experience prior to using AMASS** in order to generate a report of cumulative antibiograms?

	Scale of the problem				
	Very major	Major	Medium	Minor	No problem
The number of staff is limited, and data analysis takes a lot of time.	○	○	○	○	○
There are no staff members who can use statistical software.	○	○	○	○	○
There are no computers or laptops available for data analysis.	○	○	○	○	○
Data cannot be exported or combined into a single dataset.	○	○	○	○	○
Data is too big to analyze.	○	○	○	○	○
Data is too complicated to analyze	○	○	○	○	○
There are no automated functions in the main laboratory software or analytical software (including WHONET) to generate a "complete" cumulative antibiograms report.	○	○	○	○	○
There are no automated functions in the main laboratory software or analytical software (including WHONET) to generate "individual" cumulative antibiogram.	○	○	○	○	○
Importing data into in an analytical software (including WHONET) is very complicated.	○	○	○	○	○
Failure to import into an analytical software (including WHONET)	○	○	○	○	○
Data analysis using the main laboratory software or analytical software (including WHONET) is very difficult.	○	○	○	○	○
Data analysis using the main laboratory software or analytical software (including WHONET) is very time consuming.	○	○	○	○	○

5.6 **Other issues you encountered while generating your Cumulative Antibiogram.**

*** We understand that although you were trained to utilize AMASS to automatically generate a cumulative antibiogram report during the workshop organized by the Division of Health Administration, MoPH, **many hospitals may still have some problems when using AMASS. Your answers will help the MoPH understand the real-world problems of using AMASS, enabling them to support and provide solutions in the future.**

5.7 **Which problems did you experience after using AMASS** in order to generative a report of cumulative antibiograms?

	Scale of the problem				
	Very major	Major	Medium	Minor	No problem
The number of staff is limited, and the data analysis using AMASS takes a lot of time.	○	○	○	○	○

	There are no staffs who can use AMASS	○	○	○	○	○
	There are no computers or laptops available for AMASS.	○	○	○	○	○
	Data cannot be exported or combined into a single dataset.	○	○	○	○	○
	Data is too big for AMASS.	○	○	○	○	○
	Data is too complicate for AMASS	○	○	○	○	○
	There are no automated functions to generate the "complete" cumulative antibiograms report in AMASS.	○	○	○	○	○
	There are no automated functions to generate "individual" cumulative antibiogram in AMASS.	○	○	○	○	○
	Importing data into AMASS is very complicated.	○	○	○	○	○
	Failure to import into AMASS	○	○	○	○	○
	Data analysis using AMASS is very difficult.	○	○	○	○	○
	Data analysis using AMASS is very time consuming.	○	○	○	○	○
	Names of antibiotics, types of specimens and bacterial species in the dictionary files of AMASS is unclear	○	○	○	○	○
	The methods to fill names of antibiotics, types of specimens and bacterial species in the dictionary files of AMASS is too complicated	○	○	○	○	○
	The methods to validate names of antibiotics, types of specimens and bacterial species in the log files of AMASS is too complicated	○	○	○	○	○
**5.8**	**Any remarks on the answers in part 5**					
